# Synthesis and structure of ammonium bis­(malonato)borate

**DOI:** 10.1107/S2056989025007169

**Published:** 2025-08-19

**Authors:** Gokila Govindharajan, Ramachandra Raja Chidambaram, Gnanasheela Uthamaselvan, Kalaiarasi Iyathurai, Kamatchi Karthikeyan, Sampath Natarajan

**Affiliations:** a Government College for Women (Affiliated to Bharathidasan University) KumbakonamThanjavur Tamilnadu-612001 India; b Department of Physics, D. G. Government Arts College for Women (Affiliated to Annamalai University), Mayiladuthurai, Taminadu-609 001, India; c Department of Physics, Government College for Women (Affiliated to Bharathidasan University), Kumbakonam, Thanjavur, Tamilnadu-612001, India; d Department of Physics, Srinivasa Ramanujan Centre, SASTRA Deemed University, Kumbakonam, Thanjavur, Tamilnadu-612001, India; e Department of Chemistry, Chemical Biology Lab., School of Chemical and Biotechnology, SASTRA Deemed University, Thanjavur, Tamilnadu-613401, India; University of Aberdeen, United Kingdom

**Keywords:** crystal structure, ammonium, bis­(malonato)borate, Hirshfeld surface

## Abstract

In the title salt, NH_4_^+^·[B(C_3_H_2_O_4_)_2_]^−^, the boron atom is chelated by two malonate ligands in a bidentate fashion, resulting in a BO_4_ tetra­hedron with both chelate rings adopting shallow boat conformations. The extended structure features five N—H⋯O and three C—H⋯O hydrogen bonds, accounting for approximately 69.9% of the total inter­molecular inter­actions.

## Abstract 2

The title salt features BO_4_ tetra­hedra at the centre of [B(C_3_H_2_O_4_)_2_]^−^ anions.

## 1. Chemical context

The bis­(malonate)borate anion, [B(C_3_H_2_O_4_)_2_]^−^, is a tetra­hedral boron-centred complex in which two malonate ligands are bidentately coordinated *via* their carboxyl­ate oxygen atoms. This chelation results in a stable, symmetrical anion capable of forming extended hydrogen-bonded or ionic frameworks when paired with alkali metal cations such as sodium or potassium (Zviedre & Belyakov, 2007 ▸; Selvi *et al.*, 2024 ▸). In materials science, bis­(malonate)borate derivatives have attracted attention for their role in energy storage technologies. The lithium and sodium salts of this anion have been explored as polymeric ion conductors and electrolyte additives in lithium ion and sodium ion batteries, where their borate anions contribute to enhanced electrochemical stability and ionic conductivity (Mahanthappa & Weber, 2015 ▸).

In tribological applications, bis­(malonate)borate-based ionic liquids have shown excellent thermal stability and anti­wear performance, making them environmentally friendly alternatives to halogenated lubricants (Gusain & Khatri, 2015 ▸). In biological contexts, although less studied, the malonate ligands mimic natural chelators, suggesting potential applications in metal detoxification and enzyme inhibition. Furthermore, the aqueous solubility and biocompatibility of boron-containing compounds, including bis­(malonate)borates, position them as potential boron delivery agents in boron neutron capture therapy (BNCT) for cancer treatment (Järvinen *et al.*, 2023 ▸; Li *et al.*, 2025 ▸; Dymova *et al.*, 2020 ▸). These multifaceted properties underscore the growing inter­est in bis­(malonate)borate anions at the inter­section of green chemistry, energy materials and biomedical innovation. As part of our work in this area, we now describe the synthesis and structure of the title compound, NH_4_^+^[B(C_3_H_2_O_4_)_2_]^−^, (**I**).
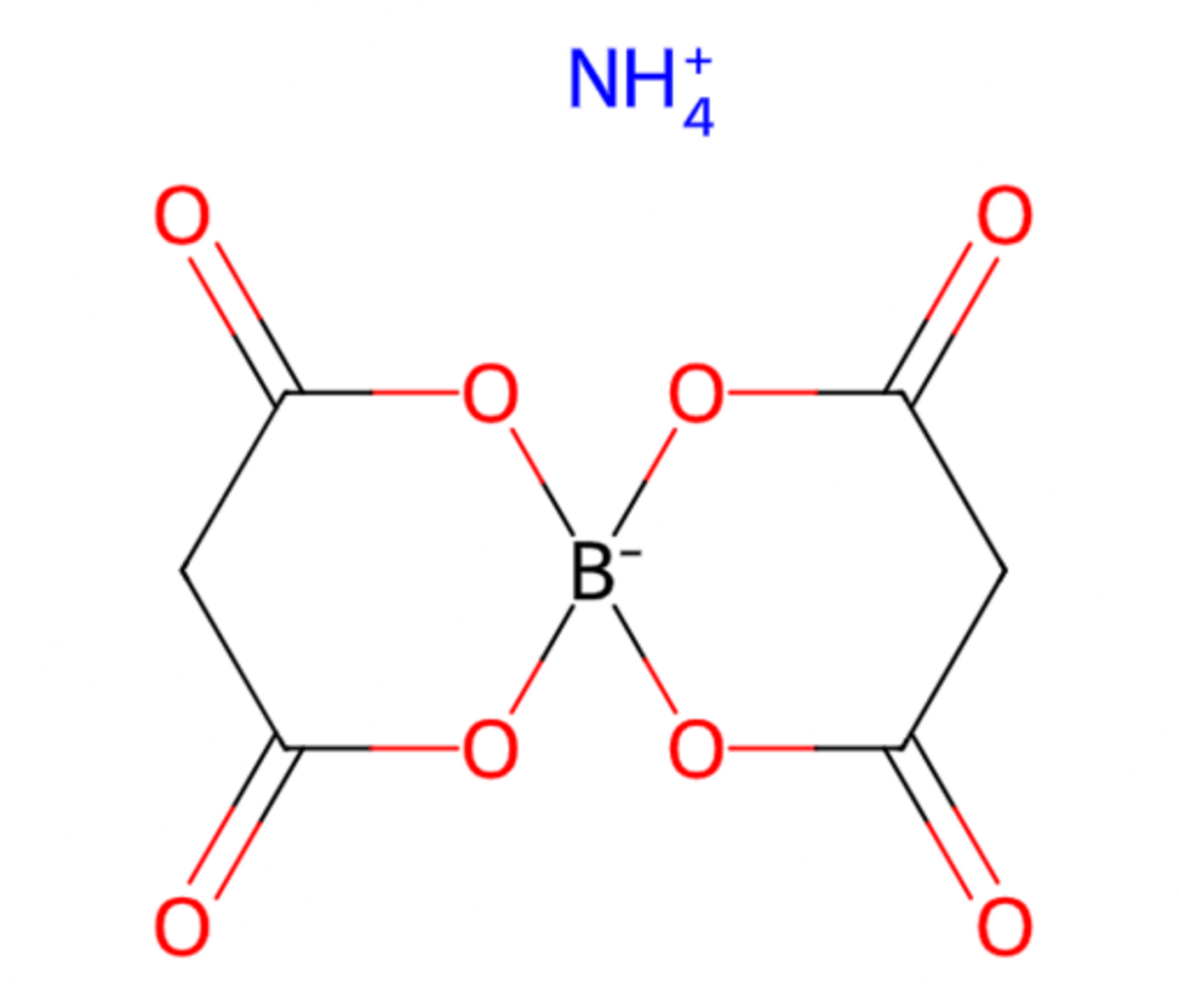


## 2. Structural commentary

**Figure 1:**
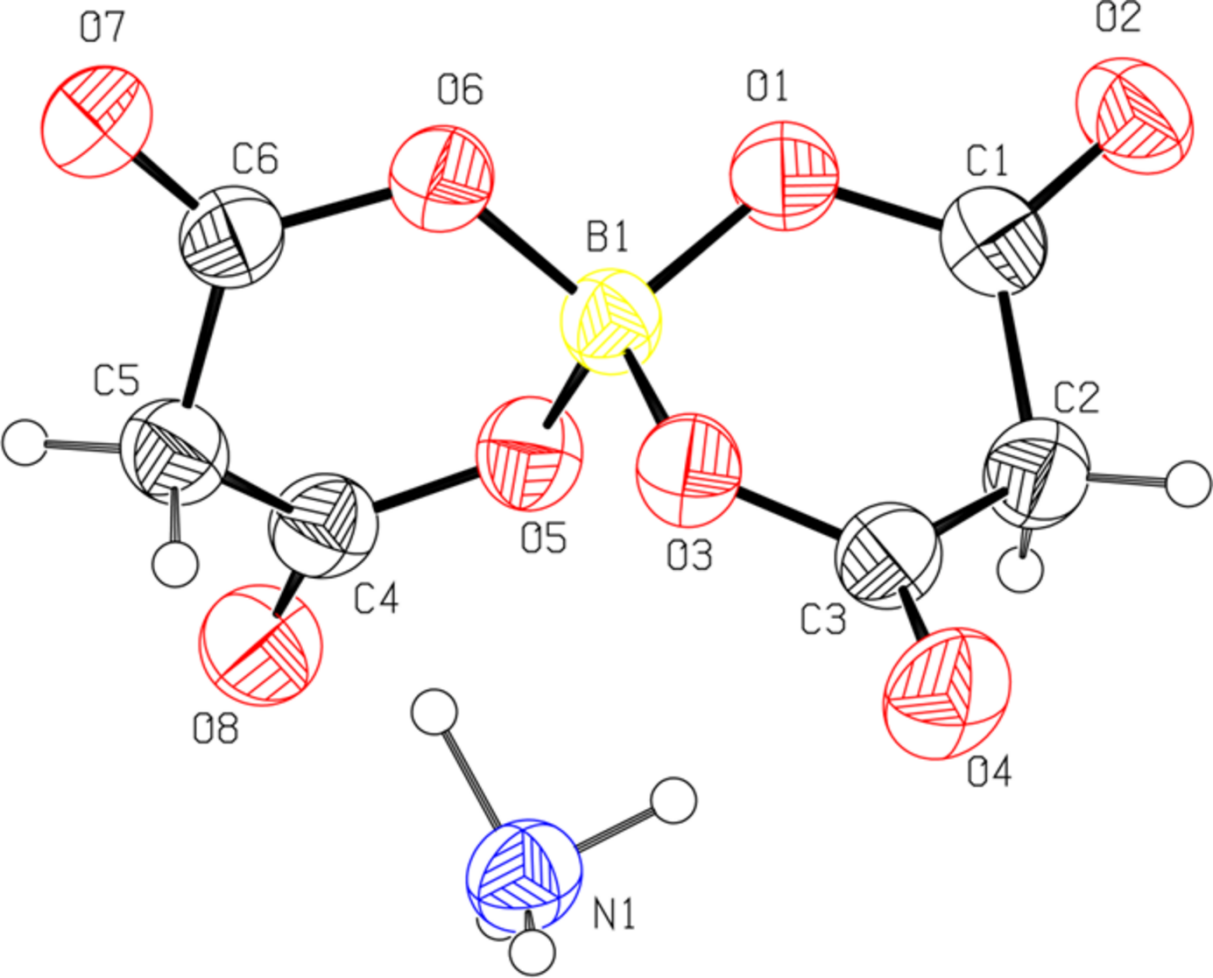
The mol­ecular structure of (**I**) showing 50% displacement ellipsoids.

**Figure 2:**
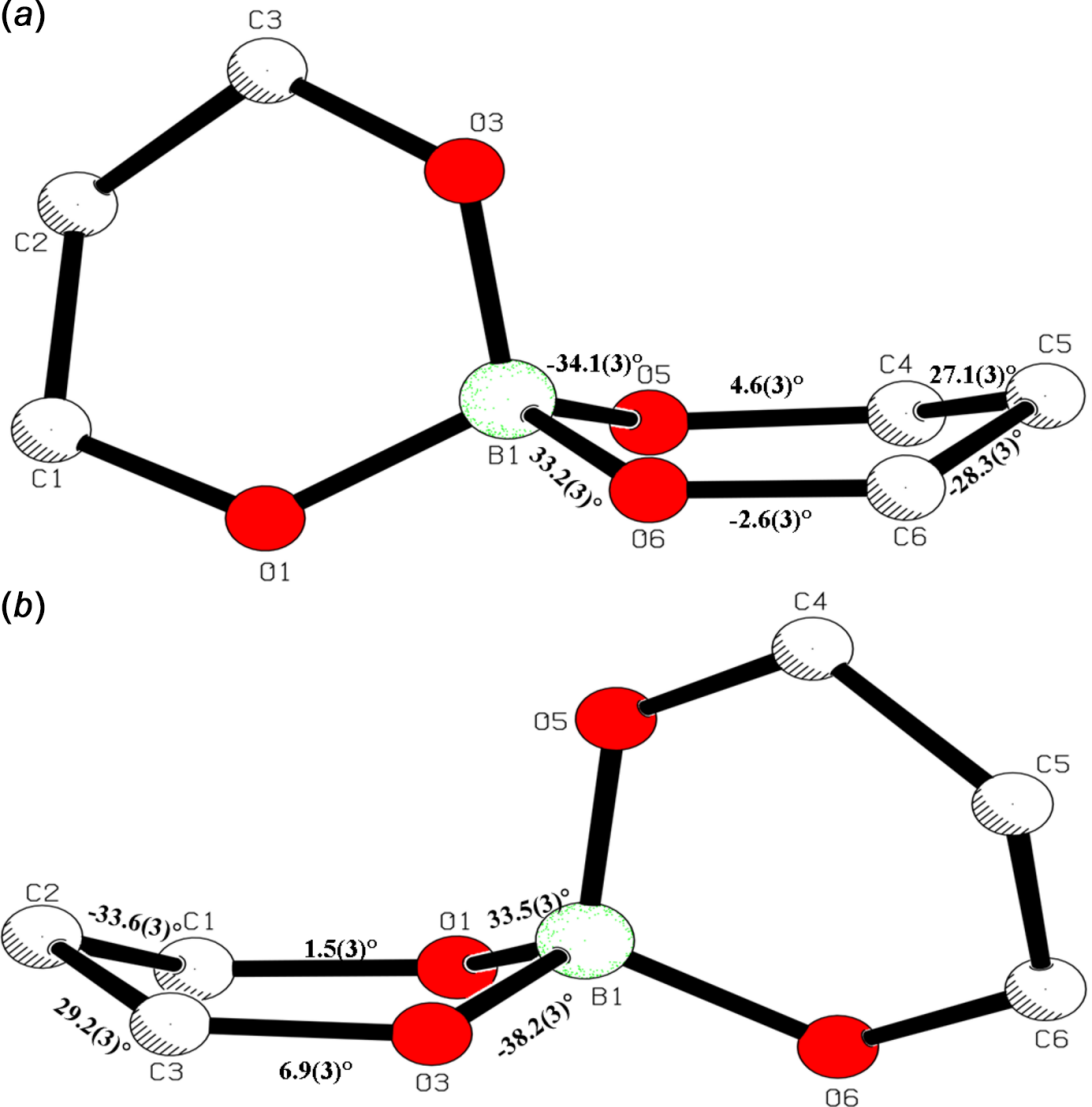
Side views of the chelate rings in (**I**) with torsion angles indicated.

Compound (**I**) features a presumed *sp*^3^-hybridized tetra­hedral B atom coordinated by two chelating malonate ligands, each binding through two carboxyl­ate O atoms (Fig. 1 ▸). Selected geometrical data are given in Table 1 ▸. In the B—O tetra­hedron, the mean B—O bond length of 1.465 Å is in good agreement with the already reported structure of sodium bis­(malonate)borate (Selvi *et al.* 2024 ▸) and also agrees with the expected value for a B*sp*^3^—O bond length (1.468 Å; Allen *et al.*, 1987 ▸). The largest O—B—O bond angles are the intra­cyclic angles: O1—B1—O3 = 112.4 (2)° and O5—B1—O6 = 112.5 (2)°. The six-membered boro–malonate rings B1/O5/C4/C5/C6/O6 (Fig. 2 ▸*a*) and B1/O1/C1/C2/C3/O3 (Fig. 2 ▸*b*) both adopt shallow boat conformations with puckering amplitudes *Q*_T_ = 0.457 (2) and 0.414 (2) Å, respectively. In the boat conformations of the boro-malonate rings (Fig. 2 ▸), atoms B1 and C2 deviate from the near planarity of other atoms (O3, C3, C1, and O1) by −0.413 (2) and −0.378 (2) Å, respectively. In the other ring, atoms B1 and C5 deviate from the mean plane of the other atoms (O5, C4, C6 and O6) by 0.386 (2) and 0.330 (2) Å, respectively. The dihedral angle between the boro-malonate rings is 76.5 (1)°, *i.e.*, they are oriented almost perpendicular to each other. The [B(C_3_H_2_O_4_)_2_]^−^ anion is charge balanced by NH_4_^+^ cations, which participate in an extensive hydrogen-bonded network.

## 3. Supra­molecular features

**Figure 3:**
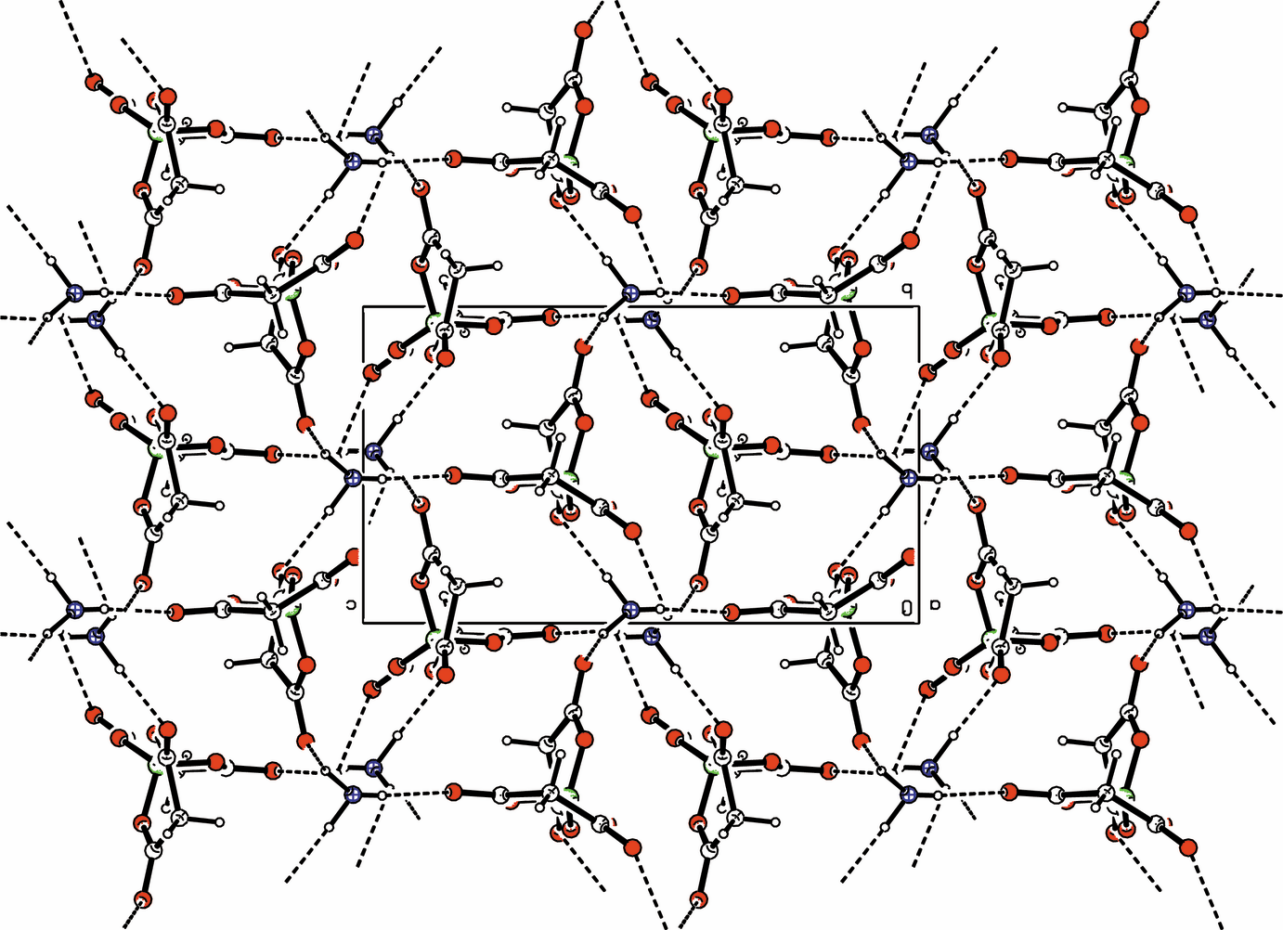
Packing diagram for (**I**) viewed down the *a*-axis direction. The dotted lines indicate the hydrogen bonds.

**Figure 4:**
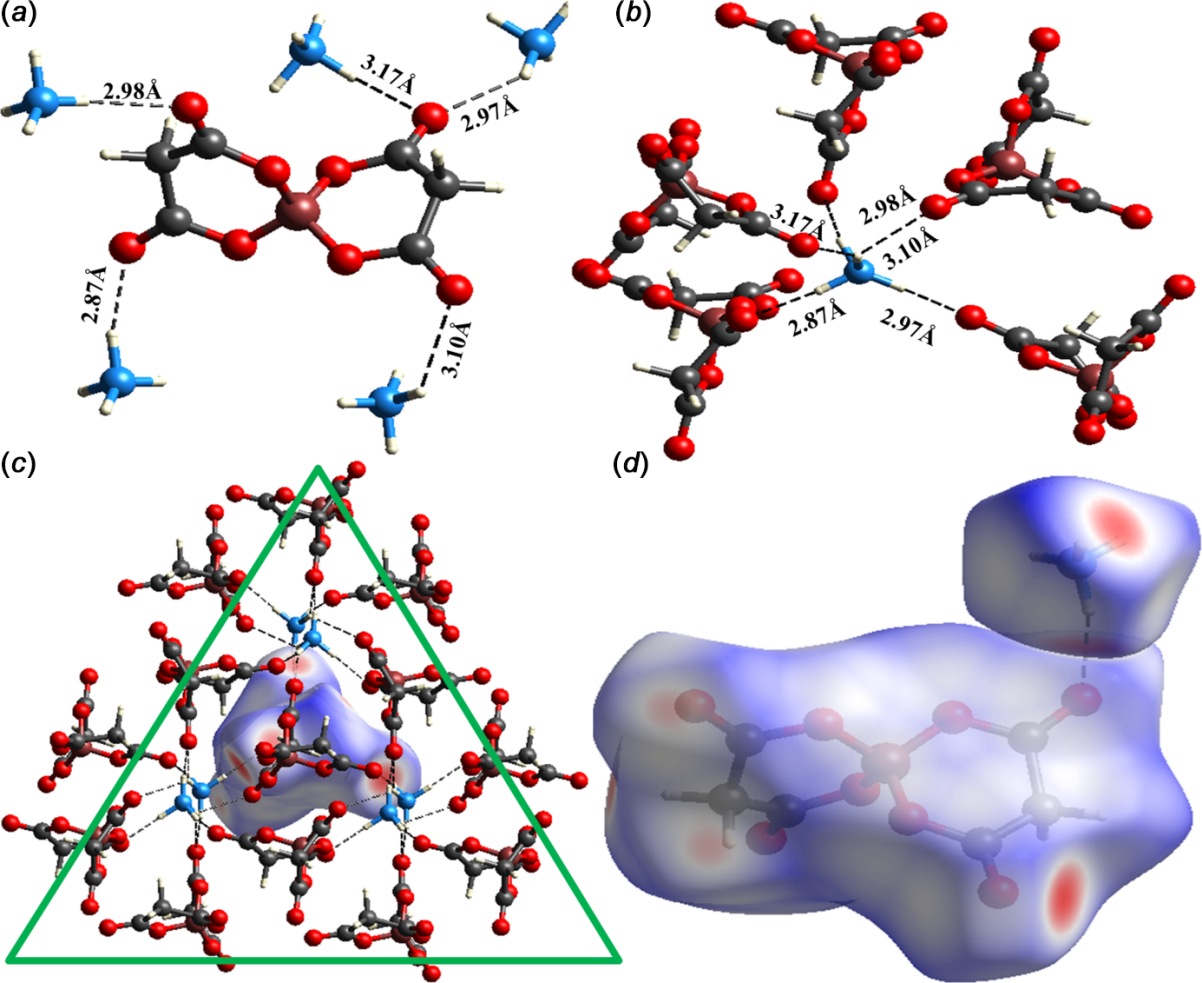
The environments of (*a*) the cation, (*b*) the anion and (*c*) the triangular supra­molecular motif in (**I**); (*d*) the Hirshfeld surface for (**I**).

The structural integrity of the extended structure of (**I**) is maintained by a network of N—H⋯O and C—H⋯O inter­actions (Fig. 3 ▸), as detailed in Table 2 ▸. Each [B(C_3_H_2_O_4_)_2_]^−^ anion accepts hydrogen bonds from five neighbouring NH_4_^+^ cations (Fig. 4 ▸*a*). Conversely, every NH_4_^+^ cation participates in analogous inter­actions with five adjacent [B(C_3_H_2_O_4_)_2_]^−^ anions (Fig. 4 ▸*b*). This results in the formation of a triangular-shaped supra­molecular assembly (Fig. 4 ▸*c*).

**Figure 5:**
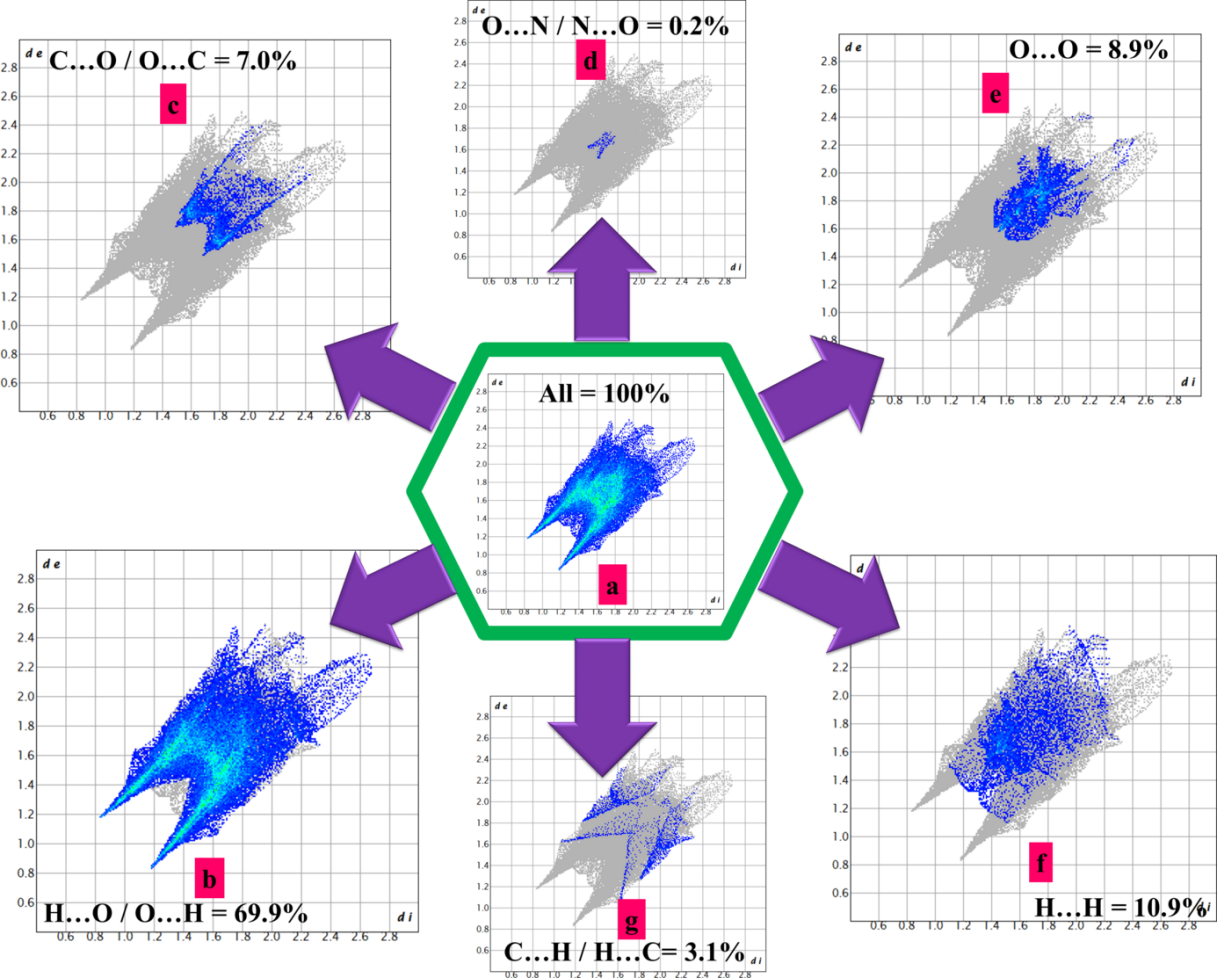
Fingerprint plots for (**I**).

Hirshfeld surface analysis of (**I**) was performed using *Crystal Explorer* (Version 21.5; Spackman *et al.*, 2021 ▸). Fig. 4 ▸*d* shows the d_*norm*_ surface for the [B(C_3_H_2_O_4_)_2_]^−^ anion where the intense red spots signify the shortest contacts (indicative of strong hydrogen bonds) and blue regions denote longer distances (suggesting weak van der Waals or repulsive inter­actions). Fig. 5 ▸ shows the two-dimensional fingerprint plots, with the overall inter­action in Fig. 5 ▸*a* and the decomposed contributions and their percentages in Fig. 5 ▸*b*–5*g*. The hydrogen bonds are distinctly marked by sharp, symmetrical wings in the H⋯O/O⋯H plot (Fig. 5 ▸*b*), which dominates the Hirshfeld surface (69.9%).

## 4. Database survey

A search of the Cambridge Structural Database (CSD 2025; Groom *et al.*, 2016 ▸) using CCDC CONQUEST revealed two related bis­(malonate)borate complexes, CSD refcode PODHAV (Selvi *et al.*, 2024 ▸) and PITQUF (Zviedre & Belyakov, 2007 ▸), featuring Na^+^ and K^+^ counter-ions, respectively. While these exhibit malanato-borate coordination geometries very similar to (**I**), they differ fundamentally through their alkali metal coordination spheres as opposed to our ammonium variant. Notably, the potassium centre in PITQUF adopts an irregular nine-coordinate geometry with oxygen donors from seven distinct anions, whereas the sodium centre in PODHAV displays an inter­mediate coordination state – primarily square pyramidal (five O-donors) but transitioning to a distorted octa­hedron upon inclusion of a sixth, more weakly bound oxygen atom.

## 5. Synthesis and crystallization

A mixture of malonic acid, boric acid, and ammonium carbonate in a molar ratio of 4:2:1 was dissolved in double-distilled water while continuously stirring. The solution was gently heated to a temperature of 313–323 K to ensure complete dissolution, resulting in a clear, homogeneous mixture. It was then allowed to cool to room temperature and was filtered to remove any particulate impurities. The filtrate was transferred to a clean glass vessel, which was covered with a perforated lid to control evaporation, and placed in a draft-free environment maintained at a temperature of 298–303 K. Over a period of 60 days, slow evaporation of the solvent led to the growth of well-defined colourless blocks of (**I**). These were carefully extracted, rinsed with cold distilled water to eliminate surface impurities, and air-dried at room temperature.

## 6. Refinement

Crystal data, data collection and structure refinement details are summarised in Table 3 ▸. The carbon-bound hydrogen atoms were positioned based on calculated values (C—H = 0.96 Å) and were refined as riding atoms, with *U*_iso_(H) set to 1.2 *U*_eq_(C). For the NH_4_^+^ ion, the hydrogen atoms were refined using DFIX restraints, maintaining an N—H distance of 0.86 Å and setting *U*_iso_(H) to 1.4*U*_eq_(N).

## Supplementary Material

Crystal structure: contains datablock(s) I. DOI: 10.1107/S2056989025007169/hb8150sup1.cif

Structure factors: contains datablock(s) I. DOI: 10.1107/S2056989025007169/hb8150Isup2.hkl

CCDC reference: 2455003

Additional supporting information:  crystallographic information; 3D view; checkCIF report

## Figures and Tables

**Table 1:** Selected geometric parameters (Å, °)

B1—O1	1.457 (3)	B1—O5	1.474 (3)
B1—O3	1.472 (3)	B1—O6	1.454 (3)
			
O1—B1—O3	112.42 (17)	O6—B1—O1	105.67 (17)
O1—B1—O5	108.36 (18)	O6—B1—O3	108.43 (18)
O3—B1—O5	109.46 (18)	O6—B1—O5	112.51 (17)

**Table 2:** Hydrogen-bond geometry (Å, °)

*D*—H⋯*A*	*D*—H	H⋯*A*	*D*⋯*A*	*D*—H⋯*A*
N1—H1*A*⋯O2^i^	0.88 (2)	2.41 (2)	3.103 (3)	137 (2)
N1—H1*A*⋯O4^ii^	0.88 (2)	2.43 (2)	3.166 (3)	142 (2)
N1—H1*B*⋯O4^iii^	0.83 (2)	2.32 (2)	2.970 (3)	136 (3)
N1—H1*C*⋯O7	0.86 (2)	2.03 (2)	2.864 (3)	165 (3)
N1—H1*D*⋯O8^iv^	0.87 (2)	2.13 (2)	2.985 (3)	168 (3)
C2—H2*A*⋯O7^v^	0.97	2.33	3.218 (3)	153
C2—H2*B*⋯O2^vi^	0.97	2.48	3.181 (3)	129
C5—H5*B*⋯O6^vii^	0.97	2.48	3.289 (3)	140

Symmetry codes: (i) 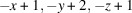; (ii) 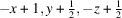; (iii) 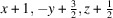; (iv) 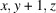; (v) 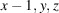; (vi) 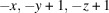; (vii) 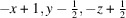.

**Table 3:** Experimental details

Crystal data	
Chemical formula	NH_4_^+^·C_6_H_4_BO_8_^−^
*M*_r_	232.94
Crystal system, space group	Monoclinic, *P*2_1_/*c*
Temperature (K)	298
*a*, *b*, *c* (Å)	9.1825 (7), 7.6234 (6), 13.6905 (10)
β (°)	102.201 (2)
*V* (Å^3^)	936.71 (12)
*Z*	4
Radiation type	Cu *K*α
μ (mm^−1^)	1.36
Crystal size (mm)	0.23 × 0.16 × 0.13

Data collection	
Diffractometer	Bruker D8 VENTURE
Absorption correction	Multi-scan (*SADABS*; Krause *et al.*, 2015 ▸)
*T*_min_, *T*_max_	0.526, 0.753
No. of measured, independent and observed [*I* > 2σ(*I*)] reflections	14514, 1771, 1631
*R*_int_	0.060
(sin θ/λ)_max_ (Å^−1^)	0.610

Refinement	
*R*[*F*^2^ > 2σ(*F*^2^)], *wR*(*F*^2^), *S*	0.066, 0.211, 1.15
No. of reflections	1771
No. of parameters	158
No. of restraints	10
H-atom treatment	H atoms treated by a mixture of independent and constrained refinement
Δρ_max_, Δρ_min_ (e Å^−3^)	0.22, −0.27

Computer programs: *APEX4* and *SAINT* (Bruker, 2021 ▸), *SHELXT2018/2* (Sheldrick, 2015*a* ▸), *SHELXL2019/3* (Sheldrick, 2015*b* ▸) and *OLEX2* (Dolomanov *et al.*, 2009 ▸).

## supplementary crystallographic information

### Ammonium bis(malonato)borate Crystal data

**Table:**

NH_4_^+^·C_6_H_4_BO_8_^−^	*F*(000) = 480
*M**_r_* = 232.94	*D*_x_ = 1.652 Mg m^−^^3^
Monoclinic, *P*2_1_/*c*	Cu *K*α radiation, λ = 1.54178 Å
*a* = 9.1825 (7) Å	Cell parameters from 9961 reflections
*b* = 7.6234 (6) Å	θ = 4.9–70.2°
*c* = 13.6905 (10) Å	µ = 1.36 mm^−^^1^
β = 102.201 (2)°	*T* = 298 K
*V* = 936.71 (12) Å^3^	Block, colourless
*Z* = 4	0.23 × 0.16 × 0.13 mm

### Ammonium bis(malonato)borate Data collection

**Table:**

Bruker D8 VENTURE diffractometer	1631 reflections with *I* > 2σ(*I*)
ω and phi scans	*R*_int_ = 0.060
Absorption correction: multi-scan (SADABS; Krause *et al.*, 2015)	θ_max_ = 70.2°, θ_min_ = 4.9°
*T*_min_ = 0.526, *T*_max_ = 0.753	*h* = −11→10
14514 measured reflections	*k* = −9→9
1771 independent reflections	*l* = −16→16

### Ammonium bis(malonato)borate Refinement

**Table:**

Refinement on *F*^2^	Hydrogen site location: mixed
Least-squares matrix: full	H atoms treated by a mixture of independent and constrained refinement
*R*[*F*^2^ > 2σ(*F*^2^)] = 0.066	*w* = 1/[σ^2^(*F*_o_^2^) + (0.1464*P*)^2^ + 0.136*P*] where *P* = (*F*_o_^2^ + 2*F*_c_^2^)/3
*wR*(*F*^2^) = 0.211	(Δ/σ)_max_ < 0.001
*S* = 1.15	Δρ_max_ = 0.22 e Å^−^^3^
1771 reflections	Δρ_min_ = −0.27 e Å^−^^3^
158 parameters	Extinction correction: *SHELXL2019/3* (Sheldrick, 2015b), Fc^*^=kFc[1+0.001xFc^2^λ^3^/sin(2θ)]^-1/4^
10 restraints	Extinction coefficient: 0.027 (5)
Primary atom site location: dual	

### Ammonium bis(malonato)borate Special details

**Table:**

Geometry. All esds (except the esd in the dihedral angle between two l.s. planes) are estimated using the full covariance matrix. The cell esds are taken into account individually in the estimation of esds in distances, angles and torsion angles; correlations between esds in cell parameters are only used when they are defined by crystal symmetry. An approximate (isotropic) treatment of cell esds is used for estimating esds involving l.s. planes.

### Ammonium bis(malonato)borate Fractional atomic coordinates and isotropic or equivalent isotropic displacement parameters (Å^2^)

**Table:**

	*x*	*y*	*z*	*U*_iso_*/*U*_eq_	
B1	0.3161 (3)	0.5528 (3)	0.36828 (18)	0.0533 (6)	
C1	0.0934 (2)	0.6346 (3)	0.42603 (16)	0.0555 (6)	
C2	0.0119 (3)	0.5359 (3)	0.33643 (18)	0.0613 (6)	
H2A	−0.088477	0.582327	0.317487	0.074*	
H2B	0.004154	0.414022	0.354991	0.074*	
C3	0.0825 (2)	0.5434 (3)	0.24711 (16)	0.0558 (6)	
C4	0.4578 (2)	0.2814 (3)	0.37743 (15)	0.0557 (6)	
C5	0.5646 (3)	0.3847 (3)	0.33053 (17)	0.0577 (6)	
H5A	0.663211	0.334372	0.352036	0.069*	
H5B	0.536011	0.370650	0.258602	0.069*	
C6	0.5738 (2)	0.5760 (3)	0.35371 (15)	0.0540 (6)	
O1	0.24075 (17)	0.6373 (2)	0.43926 (11)	0.0607 (5)	
O2	0.0333 (2)	0.7096 (2)	0.48443 (13)	0.0699 (6)	
O3	0.22873 (16)	0.5613 (2)	0.26507 (10)	0.0567 (5)	
O4	0.0112 (2)	0.5335 (3)	0.16249 (13)	0.0808 (7)	
O5	0.34322 (16)	0.3679 (2)	0.39802 (11)	0.0580 (5)	
O6	0.45349 (16)	0.6506 (2)	0.37350 (12)	0.0587 (5)	
O7	0.68341 (18)	0.6631 (2)	0.35204 (15)	0.0691 (6)	
O8	0.47475 (19)	0.1267 (2)	0.39622 (13)	0.0702 (6)	
N1	0.7675 (2)	0.9580 (2)	0.48129 (16)	0.0622 (6)	
H1A	0.833 (3)	1.024 (3)	0.461 (2)	0.093*	
H1B	0.792 (3)	0.944 (4)	0.5427 (12)	0.093*	
H1C	0.759 (3)	0.863 (3)	0.4469 (19)	0.093*	
H1D	0.683 (2)	1.013 (3)	0.465 (2)	0.093*	

### Ammonium bis(malonato)borate Atomic displacement parameters (Å^2^)

**Table:**

	*U*^11^	*U*^22^	*U*^33^	*U*^12^	*U*^13^	*U*^23^
B1	0.0510 (12)	0.0578 (13)	0.0505 (12)	0.0042 (9)	0.0090 (9)	−0.0001 (9)
C1	0.0598 (12)	0.0537 (11)	0.0550 (12)	0.0032 (8)	0.0166 (9)	0.0023 (8)
C2	0.0538 (12)	0.0726 (14)	0.0583 (13)	−0.0037 (9)	0.0138 (10)	−0.0060 (9)
C3	0.0547 (11)	0.0614 (13)	0.0482 (11)	0.0031 (8)	0.0042 (9)	−0.0001 (8)
C4	0.0569 (12)	0.0572 (12)	0.0500 (11)	0.0025 (8)	0.0046 (8)	0.0003 (8)
C5	0.0611 (12)	0.0578 (12)	0.0553 (12)	0.0022 (9)	0.0145 (9)	−0.0041 (9)
C6	0.0519 (11)	0.0608 (12)	0.0470 (10)	0.0009 (9)	0.0053 (8)	−0.0031 (8)
O1	0.0577 (9)	0.0739 (11)	0.0494 (9)	0.0029 (7)	0.0088 (7)	−0.0083 (6)
O2	0.0768 (11)	0.0700 (11)	0.0693 (11)	0.0040 (8)	0.0301 (9)	−0.0108 (7)
O3	0.0542 (9)	0.0702 (10)	0.0463 (9)	0.0030 (6)	0.0117 (6)	0.0019 (6)
O4	0.0700 (11)	0.1141 (16)	0.0515 (10)	−0.0003 (10)	−0.0026 (8)	−0.0018 (9)
O5	0.0552 (9)	0.0586 (9)	0.0601 (9)	0.0022 (6)	0.0121 (7)	0.0055 (6)
O6	0.0523 (9)	0.0552 (9)	0.0675 (10)	0.0009 (6)	0.0103 (7)	−0.0031 (6)
O7	0.0554 (10)	0.0697 (11)	0.0822 (12)	−0.0094 (7)	0.0150 (8)	−0.0105 (8)
O8	0.0737 (11)	0.0561 (10)	0.0786 (12)	0.0055 (7)	0.0110 (9)	0.0073 (7)
N1	0.0591 (11)	0.0613 (11)	0.0629 (12)	0.0001 (8)	0.0058 (9)	−0.0016 (8)

### Ammonium bis(malonato)borate Geometric parameters (Å, º)

**Table:**

B1—O1	1.457 (3)	C4—C5	1.503 (3)
B1—O3	1.472 (3)	C4—O5	1.321 (3)
B1—O5	1.474 (3)	C4—O8	1.211 (3)
B1—O6	1.454 (3)	C5—H5A	0.9700
C1—C2	1.497 (3)	C5—H5B	0.9700
C1—O1	1.327 (3)	C5—C6	1.491 (3)
C1—O2	1.207 (3)	C6—O6	1.321 (3)
C2—H2A	0.9700	C6—O7	1.210 (3)
C2—H2B	0.9700	N1—H1A	0.875 (15)
C2—C3	1.502 (3)	N1—H1B	0.830 (15)
C3—O3	1.320 (3)	N1—H1C	0.859 (15)
C3—O4	1.207 (3)	N1—H1D	0.867 (15)
			
O1—B1—O3	112.42 (17)	O8—C4—O5	120.9 (2)
O1—B1—O5	108.36 (18)	C4—C5—H5A	108.3
O3—B1—O5	109.46 (18)	C4—C5—H5B	108.3
O6—B1—O1	105.67 (17)	H5A—C5—H5B	107.4
O6—B1—O3	108.43 (18)	C6—C5—C4	115.73 (19)
O6—B1—O5	112.51 (17)	C6—C5—H5A	108.3
O1—C1—C2	116.00 (18)	C6—C5—H5B	108.3
O2—C1—C2	124.1 (2)	O6—C6—C5	116.89 (19)
O2—C1—O1	119.9 (2)	O7—C6—C5	122.9 (2)
C1—C2—H2A	108.5	O7—C6—O6	120.2 (2)
C1—C2—H2B	108.5	C1—O1—B1	121.11 (17)
C1—C2—C3	114.89 (19)	C3—O3—B1	120.08 (17)
H2A—C2—H2B	107.5	C4—O5—B1	120.90 (18)
C3—C2—H2A	108.5	C6—O6—B1	121.61 (18)
C3—C2—H2B	108.5	H1A—N1—H1B	110 (2)
O3—C3—C2	116.73 (18)	H1A—N1—H1C	108 (2)
O4—C3—C2	122.6 (2)	H1A—N1—H1D	106 (2)
O4—C3—O3	120.6 (2)	H1B—N1—H1C	115 (2)
O5—C4—C5	116.80 (19)	H1B—N1—H1D	111 (2)
O8—C4—C5	122.3 (2)	H1C—N1—H1D	107 (2)
			
C1—C2—C3—O3	29.3 (3)	O3—B1—O1—C1	33.4 (3)
C1—C2—C3—O4	−150.8 (2)	O3—B1—O5—C4	86.0 (2)
C2—C1—O1—B1	1.7 (3)	O3—B1—O6—C6	−87.6 (2)
C2—C3—O3—B1	6.9 (3)	O4—C3—O3—B1	−172.9 (2)
C4—C5—C6—O6	−28.2 (3)	O5—B1—O1—C1	−87.7 (2)
C4—C5—C6—O7	154.0 (2)	O5—B1—O3—C3	82.4 (2)
C5—C4—O5—B1	5.0 (3)	O5—B1—O6—C6	33.6 (3)
C5—C6—O6—B1	−2.9 (3)	O5—C4—C5—C6	26.9 (3)
O1—B1—O3—C3	−38.1 (3)	O6—B1—O1—C1	151.48 (19)
O1—B1—O5—C4	−151.11 (17)	O6—B1—O3—C3	−154.56 (18)
O1—B1—O6—C6	151.66 (19)	O6—B1—O5—C4	−34.7 (3)
O1—C1—C2—C3	−33.8 (3)	O7—C6—O6—B1	175.0 (2)
O2—C1—C2—C3	145.9 (2)	O8—C4—C5—C6	−152.8 (2)
O2—C1—O1—B1	−178.0 (2)	O8—C4—O5—B1	−175.3 (2)

### Ammonium bis(malonato)borate Hydrogen-bond geometry (Å, º)

**Table:**

*D*—H···*A*	*D*—H	H···*A*	*D*···*A*	*D*—H···*A*
N1—H1*A*···O2^i^	0.88 (2)	2.41 (2)	3.103 (3)	137 (2)
N1—H1*A*···O4^ii^	0.88 (2)	2.43 (2)	3.166 (3)	142 (2)
N1—H1*B*···O4^iii^	0.83 (2)	2.32 (2)	2.970 (3)	136 (3)
N1—H1*C*···O7	0.86 (2)	2.03 (2)	2.864 (3)	165 (3)
N1—H1*D*···O8^iv^	0.87 (2)	2.13 (2)	2.985 (3)	168 (3)
C2—H2*A*···O7^v^	0.97	2.33	3.218 (3)	153
C2—H2*B*···O2^vi^	0.97	2.48	3.181 (3)	129
C5—H5*B*···O6^vii^	0.97	2.48	3.289 (3)	140

Symmetry codes: (i) −*x*+1, −*y*+2, −*z*+1; (ii) −*x*+1, *y*+1/2, −*z*+1/2; (iii) *x*+1, −*y*+3/2, *z*+1/2; (iv) *x*, *y*+1, *z*; (v) *x*−1, *y*, *z*; (vi) −*x*, −*y*+1, −*z*+1; (vii) −*x*+1, *y*−1/2, −*z*+1/2.
